# A Rare Case of Tamponade Without Myocardial Rupture Following a Subacute Infarction

**DOI:** 10.7759/cureus.95447

**Published:** 2025-10-26

**Authors:** Valerie Kekenbosch, Charles Massinon, Didier Chamart, Ludovic Lefrancq, Malvine Vogel

**Affiliations:** 1 Emergency Medicine, Centre Hospitalier Universitaire (CHU) Helora Kennedy, Mons, BEL

**Keywords:** cardiovascular risk, echocardiography of the heart, heart tamponade, myocardial infarction complication, obstructive cardiogenic shock

## Abstract

Cardiac tamponade is a rare but serious complication of myocardial infarction, most often occurring in cases of complete myocardial rupture. Atypical forms, such as oozing-type rupture, without macroscopic rupture, can also cause a tamponade. We report an exceptional case of hemorrhagic tamponade due to subacute myocardial infarction without obvious myocardial rupture, occurring in a patient with no known medical history.

We report a case of a 70-year-old man who was found in shock on a public street after several weeks of atypical chest pain. On admission, he presented with cardiogenic shock and signs of peripheral hypoperfusion, severe hypotension, and tachycardia. Emergency echocardiography revealed a large circumferential pericardial effusion associated with global left ventricular hypokinesia. Biomarkers showed severe myocardial injury. CT angiography, performed before drainage to exclude aortic dissection requiring emergency surgery, confirmed the hemorrhagic effusion without dissection. While pericardial drainage was planned, the patient experienced refractory cardiac arrest. Salvage thoracotomy revealed a hemorrhagic effusion without macroscopic myocardial rupture. Post-infarction hemorrhagic tamponade may result from complete myocardial rupture (blowout) or from an “oozing” form, related to micro-tears or epicardial vascular fragility. In the present case, the rapid progression to terminal shock contrasts with previously reported cases in the literature, which were generally more subacute. Echocardiography provided an immediate diagnostic orientation, demonstrating its crucial role in managing shock. This case illustrates a rare but lethal complication of subacute myocardial infarction, i.e., hemorrhagic tamponade without complete myocardial rupture. It shows the importance of early tamponade diagnosis, the important contribution of emergency echocardiography, and the severity of silent-evolving infarctions in the context of unrecognized atherosclerotic disease.

## Introduction

Hemorrhagic cardiac tamponade is a rare (1.2%, according to the Acute Coronary Syndrome Israeli Survey 2000-2013 Registry Database) but lethal complication of myocardial infarction [1], most often associated with complete myocardial rupture [2-4]. However, atypical forms, recently described as “oozing” ruptures - micro-tears causing slow hemorrhagic leakage from myocardial muscle - may also be responsible. We report a documented case of fulminant hemorrhagic tamponade after subacute myocardial infarction without macroscopic rupture, presenting with mixed cardiogenic and obstructive shock. The present case illustrates both the potential severity of silent-evolving infarctions and the crucial role of early echocardiography in determining the etiology of shock of unknown origin.

## Case presentation

We report a case of a 70-year-old man with no known medical history or chronic treatment, found in shock on a public street. He had initially experienced atypical chest pain over several weeks but had never sought medical attention. While heading spontaneously to the emergency department, he collapsed, prompting emergency medical service intervention.

On prehospital assessment, he presented with cyanosis, peripheral mottling, and severe hypotension (70/50 mmHg). A peripheral venous line was placed without further prehospital intervention. On arrival to the emergency department, he was conscious and oriented, with a regular heart rate of 140 bpm, blood pressure of 79/40 mmHg, tachypnea with marked dyspnea, central cyanosis, and a grayish complexion. Peripheral pulses were palpable but thready, capillary refill time was >3 seconds, and jugular venous distension was noted. Chest and abdominal examination were unremarkable, with no signs of right-sided congestion or peripheral edema.

**Figure 1:**
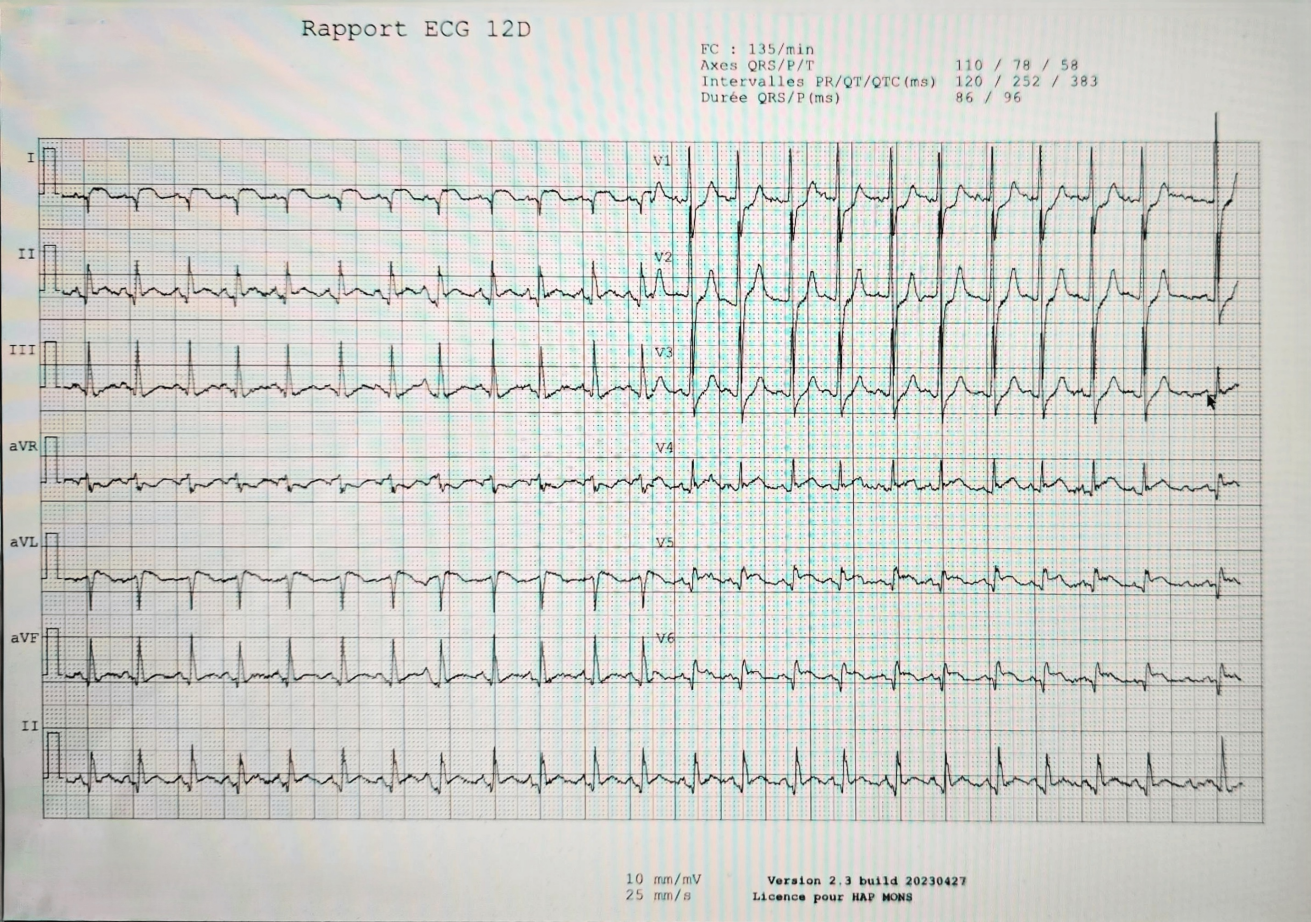
EKG at the time of admission. EKG shows a rapid sinus rhythm at 135 bpm, with ST-segment elevation in leads I, aVL, V5-V6, and ST-segment depression in aVR, without low voltage.

**Figure 2:**
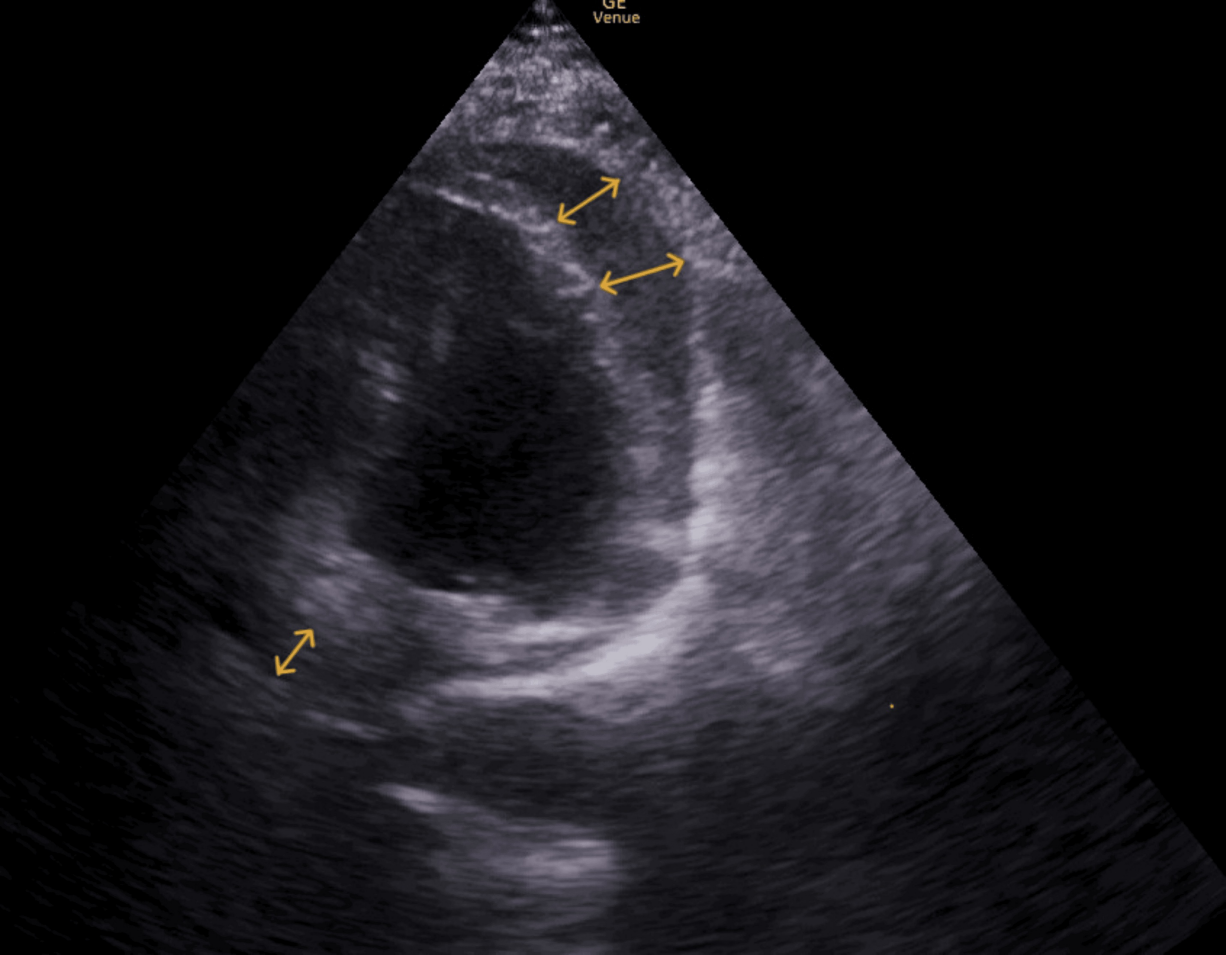
Four-chamber cardiac ultrasound. Cardiac POCUS clearly shows the significant pericardial effusion (arrows). POCUS: point-of-care ultrasound

The initial ECG showed a rapid sinus rhythm at 135 bpm, with ST-segment elevation in leads I, aVL, V5-V6, and ST-segment depression in aVR, without low voltage (Figure 1). Urgent bedside echocardiography revealed a large circumferential pericardial effusion associated with global left ventricular hypokinesia (Figure 2). Arterial blood gases showed an initially compensated lactic metabolic acidosis evolving into an uncompensated mixed metabolic and respiratory acidosis (Table 1).

**Table 1:** Geometry at admission and after cardiac arrest of the patient. pCO_2_: partial pressure of arterial carbon dioxide; pO_2_: partial pressure of arterial oxygen; HCO_3_: arterial bicarbonate concentration; BE: base excess

Parameters	Patient admission value	Patient peri-cardiac arrest value	Reference range
pH	7.42	7.01	7.35-7.45
pCO_2_ (mmHg)	12	51	35-45
pO_2_ (mmHg)	115	12	75-100
HCO_3^-^_ (mEq/L)	7.8	12.9	22-28
BE (mM/L)	-13.3	-18.2	-2 to 3
Lactate (mM/L)	8.3	10.6	0.5-2.0

Laboratory tests revealed leukocytosis, neutrophil predominance, increased inflammatory parameters, markedly elevated high-sensitivity troponin I, and kidney function impairment, with normal electrolytes and coagulation (Table 2).

**Table 2:** Patient laboratory values at the time of admission. WBC: white blood cell; US troponin I: ultrasensitive troponin I; INR: international normalized ratio

Parameters	Patient value	Reference range
WBC (×10^3^/µL)	14.38	3.8-9.8
Neutrophil (%)	75.90	38-73
C-reactive protein (mg/dL)	353.8	<0.5
US troponin I (ng/L)	26490	0-34.2
Creatinine (mg/dL)	1.89	0.72-1.25
Urea (mg/dL)	60	18-55
Sodium (mM/L)	136	136-145
Potassium (mM/L)	3.92	3.5-5.1
Chlorine (mM/L)	101	98-107
INR	1	0.8-1.2

**Figure 3:**
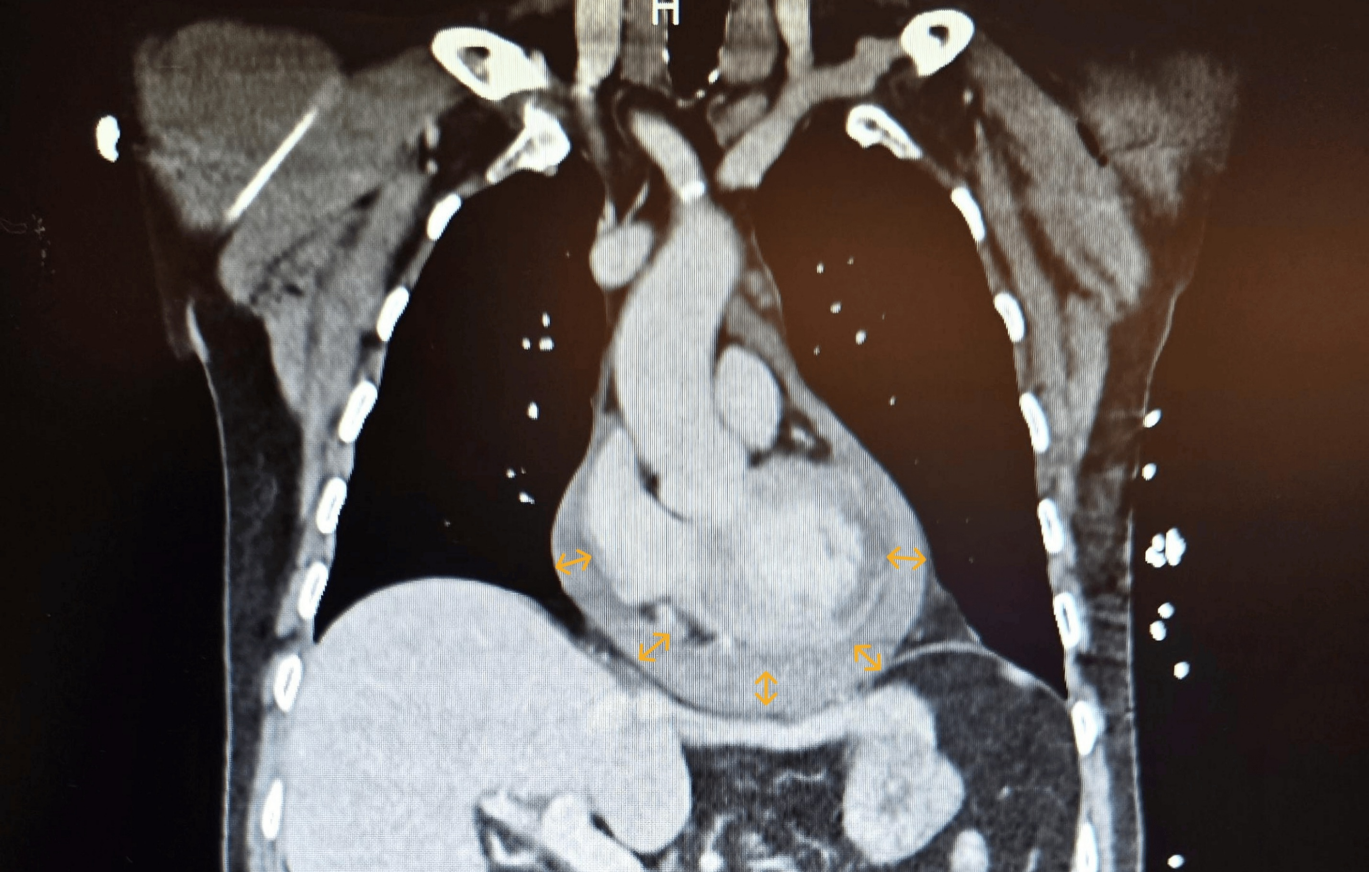
Frontal section of chest CT scan. Yellow arrows show pericardial effusion.

Emergency thoracoabdominopelvic CT angiography excluded traumatic lesions from a possible fall and aortic dissection (Figure 3). It confirmed the circumferential pericardial effusion (11 mm, blood density). It revealed an old ischemic lesion of the left medulla oblongata, atherosclerotic plaques in the left subclavian artery, early stenosis of the superior mesenteric artery, right renal atheromatosis, and coronary calcifications, consistent with previously undiagnosed diffuse atherosclerosis.

The patient was transferred to the catheterization lab for pericardial drainage. Shortly before the procedure, he developed cardiac arrest with pulseless electrical activity, quickly progressing to asystole. Cardiopulmonary resuscitation was initiated immediately and continued for 15 minutes without return of spontaneous circulation. After a non-productive drainage, salvage thoracotomy was performed by an on-site cardiac surgeon, revealing a hemorrhagic pericardial effusion and a semi-recent myocardial infarction. Despite resuscitation efforts, the patient died. Post-mortem cardiac inspection confirmed that the myocardial wall was globally intact.

## Discussion

Pericardial effusion is a known complication of myocardial infarction, particularly in extensive, non-reperfused transmuralized forms. Various etiologies should be considered, such as pericardial inflammatory reaction (early epicardial pericarditis or Dressler syndrome) [2], hemorrhagic extravasation due to necrotic inflammation of the epicardium, or, more classically, myocardial rupture [3].

Cardiac tamponade is a rare complication following myocardial infarction, occurring in approximately 1.4% of cases in the Shock trial by Hochman et al. [4]. A combination of clinical, biological, electrocardiographic, and echographic criteria leads us toward this diagnosis (table in the appendix). It occurs when a rapid rise in intrapericardial pressure exceeds right heart diastolic pressures, impeding filling. This leads to reduced cardiac output, severe hypotension, paradoxical pulse, jugular venous distension, prolonged capillary refill time, and sometimes pulseless electrical activity, as seen in our patient. The initial tachycardia and preserved consciousness reflect transient sympathetic compensation [5-7]. In the presence of a hemorrhagic tamponade, differential diagnoses include traumatic, primary or secondary tumor, vascular (type A aortic dissection), infectious, hematologic (coagulopathy, thrombocytopenia), or ischemic causes [8].

In this case, with dissection, thoracic tumor, and trauma excluded by CT, we had no reason to think of an iatrogenic cause since no procedure had yet been performed. The effusion was likely due to a severe hemorrhagic inflammatory reaction in the setting of a silent, evolving myocardial infarction. The absence of low voltage on ECG, global hypokinesia on echocardiography, and markedly elevated biomarkers (troponin >26,000 ng/L, CRP >350 mg/L, lactate >8 mmol/L) reflected major myocardial injury and mixed cardiogenic/obstructive shock. Salvage thoracotomy revealed no macroscopic myocardial rupture, though a micro-tear could not be completely excluded. Matteucci et al. recently classified myocardial ruptures into two types as follows: “blowout,” namely active bleeding with macroscopic tear, and “oozing,” namely micro-tear with slow diffuse bleeding, often from an intramyocardial hematoma [9]. The autopsy of the patient could have given us certainty, but it was not performed.

Our patient likely had the latter, presenting as a low-profile hemorrhagic leak into the pericardium. Only three other case reports describing this type of myocardial fissure were found. None of those presented in shock at admission. The rapid, refractory course in our case contrasts with the typically more subacute, better-survival presentations previously described [10-12].

This case also underscores that tamponade can complicate transmural infarction even without direct wall rupture, through epicardial vascular fragility, inflammatory neovascularization, or rupture of epicardial vessels adjacent to the infarct zone. The absence of macroscopic rupture at thoracotomy, combined with the presence of a large hemorrhagic effusion, supports the hypothesis of diffuse leakage of intramyocardial or epicardial vascular origin, related to inflammatory tissue fragility in the setting of a non-reperfused transmural infarction. This course highlights a pathophysiological mechanism that is still underrepresented in literature but potentially underdiagnosed [9].

From an echocardiographic standpoint, several findings support tamponade as the cause of shock [13]. In the present case, bedside echocardiography performed on admission allowed immediate diagnostic orientation by revealing a large circumferential pericardial effusion, inspiratory collapse of the right atrium, early diastolic collapse of the right ventricle (often early and specific), dilatation of the inferior vena cava with loss of its normal respiratory variation, and exaggerated respiratory variation in transmitral and transtricuspid flow velocities (echocardiographic equivalent of paradoxical pulse). This case highlights the pivotal role of bedside echocardiography in the emergency setting [14]. Its use enables a rapid assessment of the causes of shock (obstructive, cardiogenic, hypovolemic, or distributive) and guides major therapeutic decisions. In this instance, it allowed the diagnosis of tamponade to be made in the context of an undifferentiated shock by identifying a compressive circumferential effusion associated with global ventricular dysfunction. This imaging modality, readily available at the patient’s bedside, is essential for directing immediate management in extreme emergencies (cautious fluid resuscitation, catecholamines, pericardial drainage) and for reducing morbidity and mortality in such critical situations.

However, this is a highly operator-dependent examination [15,16]. Most studies reporting good sensitivity and specificity have been conducted by highly experienced users, which limits the generalizability of these performance results. This highlights the need for a high level of both initial and ongoing training to ensure the quality of examinations performed.

This case serves as a reminder that a silent myocardial infarction can progress to a severe mechanical complication, such as tamponade, that should be considered in the differential diagnosis of shock of unknown origin. It can be due to a mechanical rupture, particularly of the left ventricle, but also to a usually slower oozing-type form, which in this case presented in a fulminant manner.

In this case, the presentation also revealed previously unrecognized diffuse atherosclerosis, documented by CT, suggesting an infarction on a background of severe vascular disease, undetected due to the lack of medical follow-up. This context highlights the importance of cardiovascular screening and prevention, even in patients without an apparent medical history.

## Conclusions

This case illustrates a rare but lethal complication of myocardial infarction - tamponade due to hemorrhagic pericardial effusion in the absence of frank myocardial rupture. It highlights the importance of early cardiac echocardiography in rapidly identifying obstructive shock and the need to consider silent coronary syndromes in the differential diagnosis of an acute presentation of unexplained shock. This observation underscores the potential impact of unrecognized atherosclerotic disease, which can progress silently into life-threatening forms.

## Appendices

**Table 3:** Clinical, biological, electrocardiographic, and echocardiographic criteria of cardiac tamponade in myocardial infarction.

Category	Key criteria/findings
Clinical	Severe dyspnea, anxiety, agitation
	Arterial hypotension (often severe)
	Jugular venous distension
	Muffled or distant heart sounds
	Beck’s triad: hypotension + jugular venous distension + muffled heart sounds
	Pulsus paradoxus >10 mmHg
	Signs of cardiogenic shock: cold skin, mottling, oliguria
	Absence of peripheral edema (in acute onset)
Biological	Evidence of myocardial injury: elevated troponins
	Increased lactate (tissue hypoperfusion)
	Functional renal failure
	Possible anemia (if myocardial rupture with hemorrhage)
	No specific biological marker of tamponade
Electrocardiographic (ECG)	Global low voltage
	Electrical alternans (alternating QRS amplitude)
	ECG signs of myocardial infarction (ST elevation, Q waves)
	Frequent sinus tachycardia
Electrocardiographic criteria of cardiac tamponade in myocardial infarction	Circumferential pericardial effusion, sometimes compressive
	Diastolic collapse of the right ventricle
	Right atrial collapse at end-diastole
	Exaggerated respiratory variation (>25%) of mitral/tricuspid inflow
	Dilated inferior vena cava with no inspiratory collapse
	Low cardiac output signs
	Usually occurs after left ventricular free wall rupture/rarely oozing type rupture
	Sudden shock with electromechanical dissociation
